# Introduction of a penicillin allergy de-labelling program with direct oral challenge and its effects on utilization of beta-lactam antimicrobials: a multicenter retrospective parallel cohort study

**DOI:** 10.1186/s13223-024-00877-9

**Published:** 2024-03-05

**Authors:** Adhora Mir, Derek Lanoue, Veronica Zanichelli, Carl van Walraven, Timothy Olynych, Caroline Nott, Derek MacFadden

**Affiliations:** 1 The Ottawa Hospital, 501 Smyth Rd, Ottawa, ON Canada; 2 The Ottawa Hospital Research Institute, Ottawa, Canada; 3 The University of Ottawa, Ottawa, Canada; 4 Division of Allergy and Clinical Immunology, Department of Medicine, McGill University Health Centre, McGill University, Montreal, QC Canada

**Keywords:** Penicillin, Allergy, Delabel, Oral challenge, Beta lactam, Amoxicillin, Stewardship

## Abstract

**Background:**

Self-reported penicillin allergy labels are common and often inaccurate after assessment. These labels can lead to reduced use of first-line beta-lactam antibiotics and worse outcomes. We measured the impact of a previously performed inpatient proactive systematic penicillin allergy de-labelling program on subsequent antibiotic use. This prior program included assessment, risk-stratification, and low risk direct oral amoxicillin challenge.

**Methods:**

We performed a retrospective comparison of parallel cohorts from two separate tertiary care hospital campuses in Ottawa, Canada across two penicillin de-labelling intervention periods across April 15th to April 30th, 2021, and February 15th to March 8th, 2022. Outcomes, including penicillin allergy labelling and antibiotic use, were collected for the index admission and the subsequent 6-month period. Descriptive statistics and multivariate regression analyses were performed.

**Results:**

A total of 368 patients with penicillin allergy label were included across two campuses and study periods. 24 (13.8%) patients in the intervention groups had sustained penicillin allergy label removal at 30 days from admission vs. 3 (1.5%) in the non-intervention group (p < 0.001). In the 6-months following admission, beta-lactams were prescribed more frequently in the intervention groups vs. the non-intervention groups for all patients (28 [16.1%] vs.15 [7.7%], p = 0.04) and were prescribed more frequently amongst those who received at least one antibiotic (28/46 [60.9%] vs.15/40 [37.5%], p = 0.097). In a multivariate regression analysis, the intervention groups were found to be associated with an increased odds of beta-lactam prescribing in all patients (OR 2.49, 95%CI 1.29–5.02) and in those prescribed at least one antibiotic (OR 2.44, 95%CI 1.00–6.15). No drug-related adverse events were reported.

**Conclusions:**

Proactive penicillin allergy de-labelling for inpatients was associated with a reduction in penicillin allergy labels and increased utilization of beta-lactams in the subsequent 6-months.

**Supplementary Information:**

The online version contains supplementary material available at 10.1186/s13223-024-00877-9.

## Introduction

Approximately 10% of inpatients report a penicillin allergy, but studies have shown that over 90% of these patients will tolerate a penicillin-based antibiotic [1, 2]. Moreover, many patients with penicillin allergy labels are identified as having “low risk” histories including remote cutaneous only reactions and can be de-labeled safely through physician-led direct penicillin oral challenge [3]. Avoidance of penicillin-based antibiotics due to penicillin allergy labels leads to unnecessary use of second-line agents, glycopeptides, fluoroquinolones, lincosamides, or aminoglycosides, that can be less effective, have a greater risk of side effects such as *C. difficile* infection, and are often costlier than beta-lactams [4, 5]. Where there is a prescribing barrier to cephalosporins in patients with reported penicillin allergy, this effect may be greater. Therefore, penicillin de-labelling programs (where reported penicillin allergies are assessed and removed were possible) are an important antimicrobial stewardship tool by removing inaccurate penicillin allergy labels [1]. However, their uptake has been suboptimal as previous guidelines advised intradermal testing, which is more labour-intensive and requires specialist input, compared to oral testing [6].

Recent studies have demonstrated the safety and efficacy of oral challenge of penicillin-based antibiotics for inpatients with remote, low-risk, cutaneous-only reactions including urticaria and morbilliform eruption [7–13]. In a retrospective review of military recruits undergoing direct oral amoxicillin challenge, 0/328 (0%) and 5/328 (1.5%) experienced an anaphylactic or any reaction, respectively [11]. Ramsey et al. [9] and Ramsey and Mustafa [12] demonstrated the efficacy of oral challenge of penicillin-based antibiotics in the inpatient and outpatient setting in patients with low-risk, cutaneous-only reactions occurring more than 10 years ago. Confino-Cohen and colleagues challenged a total of 617 patients with a history of non-immediate reactions regardless of skin test results and only 9 patients (1.5%) experienced an immediate reaction, all of which were mild in that they were non-severe cutaneous reactions [13]. Mill et al. demonstrated an exceptional safety profile of direct challenges in the pediatric population with a history of cutaneous reactions [14]. While direct oral challenge appears safe, real-world data are needed to support the feasibility of implementation in the inpatient setting and the downstream impacts of de-labelling programs, including subsequent utilization of beta-lactam antibiotics.

We sought to measure the expected benefit of introducing a standardized de-labelling program at two large campuses of a tertiary care academic hospital, with a focus on downstream antibiotic prescribing impacts.

## Methods

### Study design

A systematic inpatient penicillin allergy de-labeling program was previously implemented at a large 1300-bed academic tertiary care center (The Ottawa Hospital) in Ottawa, Canada as part of an antimicrobial stewardship led quality improvement initiative during two separate time periods (as defined below) at two campuses (General Campus and Civic Campus). We performed a retrospective, parallel cohort study, to measure its effect on penicillin allergy de-labelling and antibiotic prescribing in the subsequent 6-month period.

**Fig. 1:**
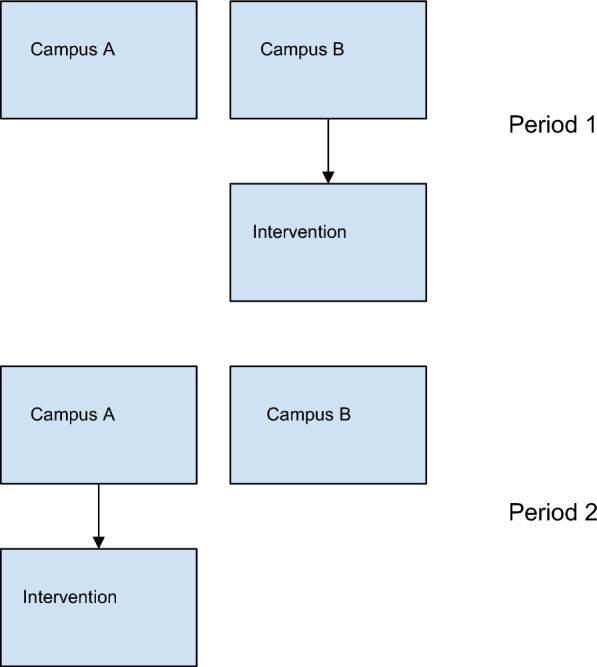
Schematic representation of the de-labelling implementation program

Data for this study was collected retrospectively from across two time periods defined as Period 1 (April 15th to April 30th, 2021), and Period 2 (February 15th to March 8th, 2022). During Period 1, the intervention occurred at Campus B but not Campus A, and during Period 2 the intervention occurred at Campus A but not Campus B (Fig. 1), creating a natural parallel cohort with the potential for balancing of patient characteristics between intervention and non-intervention periods. Ethics approval from the *Ottawa Health Science Network Research Ethics Board* was obtained for this retrospective study. The prior described quality improvement initiative had an REB exemption at the Ottawa Hospital. STROBE guidelines were followed during the development, analysis, and reporting of this observational study [15].

### The penicillin de-labelling program with oral amoxicillin challenge

A penicillin allergy de-labelling with oral amoxicillin challenge quality improvement initiative was previously implemented during two time periods at two separate campuses as previously noted. We have described this program in detail in Additional file 1: Methods section, as well as in brief herein. Inclusion criteria included all inpatients ≥ 18 years admitted for > 24 h to a medical or surgical service who had a reported penicillin allergy label listed in EPIC electronic medical record system were identified and screened by study personnel (physician). During participant screening, each patient’s record was reviewed to identify the presence of any exclusion criteria: (a) pregnancy; (b) respiratory or hemodynamic instability (SBP < 100, HR > 120, need for vasopressors, requiring > 4L/min oxygen); (c) documented history of active suicidal ideation, dementia, current delirium or admission to psychiatry ward; (d) active COVID-19 infection. The latter criterion was included for infection control reasons. Those with same-day surgical admission were excluded for the purposes of this study.

The prior intervention consisted of daily systematic screening of adult hospital inpatients admitted to medical or surgical services to identify patients with a penicillin allergy label documented in the electronic medical record (EMR; “Epic”). Patients meeting eligibility criteria were further evaluated to assess their risk of true penicillin allergy. Low-risk patients were identified and offered amoxicillin oral challenge (250 mg oral dose × 1) if they met eligibility requirements for the procedure and with approval from their primary treatment team. Some patients, with a family history of penicillin allergy but no personal history of a penicillin allergy or drug intolerance had their penicillin allergy label directly removed from their chart. Those who met the inclusion criteria and consented to oral challenge following low-risk stratification, or for whom penicillin allergy label was directly removed as above, were categorized as delabeled patients. Inaccurate penicillin allergy labels were removed from the patients’ shared electronic chart within the hospital system, as well as a letter sent to their family physician and pharmacy, to reduce the barrier for patients to receive penicillin antibiotics after successful direct oral challenge. Moderate and high-risk patients were referred to an Immunologist and Allergist for further evaluation as an outpatient. If patients could not be classified, they were reviewed at weekly meetings with investigators, including a board-certified Clinical Immunologist and Allergist (Additional file 1: Method S1), to determine appropriate allergy testing group placement (Additional file 2: Figure S1).

### Population

We modelled our study inclusion and exclusion criteria after those employed by the described proactive penicillin allergy de-labelling implementation program to identify patients that may have been reasonably intervened upon. We applied these criteria to both campuses for each of the time periods in order to capture parallel cohorts (Fig. 1), one that was receiving the program in that period and the other one that was not. We included inpatients admitted for > 24 h between April 15th 2021 to April 30th 2021 and February 15th 2022 to March 8th 2022 to a medical or surgical service at the two specified hospitals who had a reported penicillin allergy label listed in the hospital EMR (EPIC). Those admitted to a psychiatric service or with COVID-19 positive status on the first 3 days of admission were excluded. The latter criterion was included for infection control reasons.

### Outcomes

The two primary outcomes were: (1) removal of penicillin allergy label at 30 days from admission; and (2) receipt of a beta-lactam antibiotic within 6-months from initial admission amongst those who received at least one antibiotic. Secondary outcomes included: (1) presence of penicillin allergy label in the EMR at 48 h from admission (2) presence of penicillin allergy label in the EMR at 6-months from admission; (3) prescription of a beta-lactam antibiotic on initial admission amongst all patients; (4) prescription of a beta-lactam antibiotic within 6-months from initial admission amongst all patients; (5) prescription of a non-beta-lactam antibiotic on initial admission amongst all patients; (6) prescription of a non-beta-lactam antibiotic within 6-months of initial admission amongst all patients; (7) prescription of a beta-lactam antibiotic on initial admission amongst those who received at least one antibiotic; (8) prescription of a non-beta-lactam antibiotic on initial admission amongst those who received at least one antibiotic; (9) prescription of a non-beta-lactam antibiotic within 6-months from admission amongst those who received at least one antibiotic; (10) the prevalence of *C. difficile* infection by 3-months (as determined by a documented *C. difficile* stool PCR test); (11) length of stay of initial admission (Additional file 1: Method S1).

### Covariates

Covariates included: (1) demographics (age and sex); (2) admitting service (medical or surgical); (3) comorbidities via the Charlson comorbidity score [16]; (4) number of non-penicillin allergy labels; and (5) use of systemic antibiotics at our hospital within 6-months prior to admission.

### Statistical analysis

Retrospective analysis was conducted on all patients with penicillin allergy recorded within the EMR across the intervention (pro-active screening and risk stratification by investigator) and non-intervention (those without pro-active screening) groups in both study periods (Additional file 1: Method S1). Descriptive statistics were presented as counts and continuous variables, and summarized as proportions and means/medians. We compared count variables using chi-square testing and continuous variables via t-test. Descriptive statistics were stratified by relevant covariates. We used multivariable logistic regression modeling to calculate effect estimates of intervention on the primary outcome and selected secondary outcomes after adjusting for the aforementioned covariates including campus. Statistical analysis was performed using R version 4.2.1 (June 2022) and RStudio version 2022.07.2 + 576 software.

## Results

A total of 368 patients with penicillin allergy label were included across the two campuses and study periods in the retrospective analysis. For both campuses, mean age ranged from 60–61 years, and the majority of patients were female (63%-72%). Most patients were admitted under medical services (51–55%) and had more than 1 allergy label (Table 1). Sex, age, admitting service, antibiotic prescribing in the prior 6 months, number of non-penicillin allergy labels and Charlson morbidity index score were similar between intervention and non-intervention groups (Table 1).

**Table 1:** Baseline characteristics by hospital and intervention periods

	Intervention		Non-intervention		Total	
	Campus A (N = 74)	Campus B (N = 100)	Campus A (N = 107)	Campus B (N = 87)	Campus A (N = 181)	Campus B (N = 187)
Sex						
Female	50 (67.6%)	75 (75.0%)	64 (59.8%)	60 (69.0%)	114 (63.0%)	135 (72.2%)
Male	24 (32.4%)	25 (25.0%)	43 (40.2%)	27 (31.0%)	67 (37.0%)	52 (27.8%)
Age						
Mean (SD)	57.4 (21.6)	61.5 (20.2)	62.3 (17.6)	59.8 (18.7)	60.3 (19.4)	60.7 (19.5)
Charlson score						
Mean (SD)	1.04 (1.50)	1.63 (2.59)	1.32 (1.81)	1.67 (2.55)	1.20 (1.69)	1.65 (2.56)
Admitting service						
Medical	38 (51.4%)	49 (49.0%)	54 (50.5%)	53 (60.9%)	92 (50.8%)	102 (54.5%)
Surgical	36 (48.6%)	51 (51.0%)	53 (49.5%)	34 (39.1%)	89 (49.2%)	85 (45.5%)
Number of other allergies						
Mean (SD)	1.18 (1.58)	1.45 (1.94)	1.51 (2.03)	1.68 (2.45)	1.38 (1.86)	1.56 (2.19)
Antibiotics used 6 months prior to admission						
Antibiotics used	14 (18.9%)	12 (12.0%)	21 (19.6%)	19 (21.8%)	35 (19.3%)	31 (16.6%)
No antibiotics used	60 (81.1%)	88 (88.0%)	86 (80.4%)	68 (78.2%)	146 (80.7%)	156 (83.4%)

Values are reported as counts (percentages) unless otherwise specified

A greater number of patients were de-labelled at 30-days in the intervention group (24 [13.8%]) compared to the non-intervention group (3 [1.5%]) (p < 0.001) (Table 2). Of the 24 patients de-labelled in the intervention arm, 19 had received a direct oral challenge and 5 were directly de-labelled based on history. No significant drug reactions were reported.

**Table 2:** Unadjusted primary outcomes by intervention periods

	Intervention (N = 174)	Non-intervention (N = 194)	Total (N = 368)	P-Value	OR (95% CI)
Patients delabeled 30 days from admission	24 (13.8%)	3 (1.5%)	27 (7.3%)	< 0.001	10.19 (3.01–34.47)
Beta-lactam use 6 months from admission^a^	28 (60.9%)	15 (37.5%)	43 (50.0%)	0.097	2.59 (1.08–6.20)

Values are reported as counts (percentages) unless otherwise specified

^a^Among those patients who received any antibiotic

During the index admission, 113 (64.9%) and 112 (57.7%) patients received an antibiotic in the intervention and non-intervention groups respectively. Among these patients, 63 (55.8%) and 64 (57.1%), received beta-lactams in the intervention and non-intervention group, respectively (p = 0.98) (Table 3).

**Table 3:** Unadjusted secondary outcomes by intervention periods

	Intervention (N = 174)	Non-intervention (N = 194)	Total (N = 368)	P-Value	OR (95% CI)
Penicillin allergy delabelling					
Patients delabeled 48 h from admission	23 (13.2%)	2 (1.0%)	25 (6.8%)	< 0.001	14.63 (3.39–62.99)
Patient delabeled 6 months from admission	26 (14.9%)	3 (1.5%)	29 (7.9%)	< 0.001	11.18 (3.32–37.67)
Prescribing amongst all patients					
Beta-lactam use during admission	63 (36.2%)	64(34.0%)	129(35.1%)	0.91	1.10 (0.72–1.69)
Beta-lactam use 6 months from admission	28 (16.1%)	15 (7.7%)	43 (11.7%)	0.04	2.29 (1.18–4.45)
Non Beta-lactam use during admission	76 (43.7%)	65 (34.5%)	143 (38.9%)	0.2	1.47 (0.96–2.24)
Non Beta-lactam use 6 months from admission	24 (14.4%)	29 (14.9%)	54 (14.7%)	0.99	0.95 (0.53–1.70)
Prescribing amongst those who received at least one antibiotic					
Beta-lactam use during admission	63 (55.75%)	64 (57.1%)	127 (56.4%)	0.98	1.49 (0.86–2.56)
Non Beta-lactam use during admission	76 (67.3%)	65 (58.0%)	141 (62.7%)	0.36	0.95 (0.56–1.60)
Non Beta-lactam use 6 months from admission	24 (52.2%)	29 (72.5%)	53 (61.6%)	0.15	0.41 (0.17–1.02)
*C. difficile* infection 3 months from admission	2 (1.1%)	4 (2.1%)	6 (1.6%)	0.79	0.55 (0.10–3.05)
Mean length of stay (SD)	6.93 (8.49)	7.74 (10.8)	7.35 (9.77)	0.42	NA

Values are reported as counts (percentages) unless otherwise specified

Of patients who received an antibiotic prescription from all eligible sites within the hospital system during the 6-months following admission, beta-lactams were prescribed more frequently in the intervention groups (28 [60.9%]) compared with the non-intervention groups (15 [37.5%]) (Table 3).

Non-beta-lactam use during initial admission among those who used antibiotics did not significantly differ between intervention and non-intervention groups, occurring in 76 (67.3%) patients in the intervention group and 65 (58%) in the non-intervention group (Table 3). Amongst those who received at least one antibiotic in the 6-months after initial admission, there were a greater proportion of patients in the non-intervention group (29 [72.5%]) receiving non-beta lactam antibiotics compared to the intervention group (24 [52.2%]), although this was not statistically significant (p = 0.15). Notably, there were no differences in the total number of patients receiving any antibiotic within 6-months between intervention (46 [26.4%]) and non-intervention (40 [20.6%]) groups.

There was no significant difference in the incidence of *C. difficile* infection at 3 months [1.2% (n = 2) and 2.1% (n = 4)] amongst the intervention and non-intervention groups, respectively.

The mean length of stay in the intervention group (6.93 days) was shorter that the mean length of stay in the non-intervention group (7.74 days), however this difference was not statistically significant (p = 0.42).

After adjusting for potential confounding factors with multivariable logistic regression, the intervention groups were found to be significantly associated with increased beta-lactam use in the 6-months following index admission amongst those who received antibiotics (OR 2.44, 95% CI 1.00–6.15, p = 0.05) (Table 4). When we expanded the analysis to all patients (not just those having received an antibiotic), the effect was more pronounced (OR 2.49, 95% CI 1.29–5.02, p = 0.008) (Table 4).

**Table 4:** Multivariable adjusted odds ratios of predictors for primary and selected secondary outcomes

	Patients delabeled 30 days from admission	Beta-lactam use 6 months from admission (antibiotic users)	Beta-lactam use 6 months from admission (all comers)
Intervention Status	12.34 (4.12–53.35)	2.44 (1.00–6.15)	2.49 (1.29, 5.02)
Campus	4.25 (1.76–11.47)	0.89 (0.34–2.30)	1.68 (0.87–3.17)
Sex	0.99 (0.39–2.68)	1.26 (0.45–3.27)	0.85 (0.42–1.75)
Age	1.00 (0.98–1.03)	1.01 (0.98–1.04)	1.00 (0.98–1.02)
Charlson	1.03 (0.82–1.37)	1.07 (0.86–1.34)	0.99 (0.86–1.18)

## Discussion

In this study we found that implementation of an inpatient proactive systematic penicillin allergy de-labelling program with oral amoxicillin challenge was associated with both increased removal of penicillin allergy labels at 30 days from admission, and a greater utilization of beta-lactams when patients were prescribed antibiotics within 6-months of the intervention period. Furthermore, there were no adverse events of the direct oral challenge reported from the original direct oral challenge intervention. Introduction of de-labelling programs in the adult inpatient setting appears safe, effective, and can help patients preferentially receive beta-lactam antibiotics, typically the first-line class of antibiotics.

Only three patients (1.5%) of those with reported penicillin allergy labels in the non-intervention group had their penicillin allergy label removed. From previous literature, this is much lower than the expected proportion of patients with histories that would qualify them as low risk and appropriate for direct oral challenge [2]. This is reflective of the low level of de-labeling based on passive assessment by the pharmacy team during medication reconciliation.

Our study complements other studies in the literature, showing the effectiveness, safety, and effect on antibiotic prescribing of an inpatient penicillin allergy assessment program [7–13]. Recent studies by Ramsey et al*.* [9] and Chua et al*.* [10] support the safety of this direct oral challenge approach, and it is the standard of care for assessment of low risk penicillin allergies outlined in the current North American drug allergy guidelines [1].

Few studies have evaluated the downstream consequences of de-labelling on antibiotic prescribing [3, 9, 17], and typically do so by evaluating only those outcomes in patients receiving the specific intervention. In this paper, we demonstrate a significant impact at the level of all patients with penicillin allergy label who were located at a hospital receiving the de-labelling intervention, and its expected benefit compared to a parallel non-intervention cohort. These findings provide compelling evidence for the broader adoption of these approaches and their potential to be implemented as a part of institutional antimicrobial stewardship programs [3].

Commensurate with the aim of improving beta-lactam usage, we did see a trend towards reduced proportional non-beta-lactam use in the intervention group compared to the non-intervention group in 6-months after admission, which fits with the expected replacement of non-beta-lactam antibiotics by beta-lactam antibiotics for those who were de-labelled. While there was no significant effect of the intervention on incidence of *C. difficile* infection in the 3 months following admission, the outcome was rare. Initial admission length of stay was not markedly impacted by the intervention, but may have a delayed impact on decreased length of stay in subsequent visits, and warrants further evaluation.

Our study has several limitations. First, the original de-labelling intervention excluded a large number of inpatients prior to assessment. Many of the potentially eligible patients were significantly unwell with 56% of patients being excluded from assessment based on pre-defined exclusion criteria. The primary reasons for exclusion were cognitive issues that would prevent accurate assessment such as delirium, dementia, or active suicidal ideation. In spite of these exclusions, we still found a marked beneficial impact of the intervention. A second limitation of the study was that it was retrospective in nature and without randomization. While this does pose some limitations to inference, the study did benefit by the ability to compare two parallel cohorts that changed intervention periods, which may yield some additional balancing of confounders beyond those measured and adjusted for in the analyses (see Additional file 3: Table S1 for covariates between intervention and non-intervention groups). We do not believe that there would be significant carry-over effects between the implementation periods.

In summary, our study supports the adoption of inpatient programs for penicillin allergy de-labelling with direct oral challenge to help reduce the number of inappropriate penicillin allergy labels in patients and enable improved future utilization of first line beta-lactam antibiotics. Future studies are needed to answer whether these interventions result in improvements across other patient outcomes including length of stay, antibiotic toxicities, and even infection-related morbidity and mortality.

### Supplementary Information

**Additional file 1: Method S1.**

**Additional file 2: Figure S1.** Risk stratification algorithm.

**Additional file 3: Table S1.** Baseline characteristics of intervention and non-intervention groups.

## Data Availability

The data and materials for this study are available upon request.

## Abbreviations

ASP: Antimicrobial Stewardship Program
DMARD: Disease modifying antirheumatic drugs
EMR: Electronic medical record
HR: Heart rate
SBP: Systolic blood pressure
TOH: The Ottawa Hospital
