# Supplementary material for: Introduction of a penicillin allergy de-labelling program with direct oral challenge and its effects on utilization of beta-lactam antimicrobials: a multicenter retrospective parallel cohort study
Source: Allergy Asthma Clin Immunol. 2024 Mar 5;20:20. doi: 10.1186/s13223-024-00877-9 (PMC10913637; doi:10.1186/s13223-024-00877-9)
Supplement: Supplementary file 1 — Additional file 1: Method S1. [file 13223_2024_877_MOESM1_ESM.docx]

**ADDITIONAL MATERIALS**

*Procedures Followed for Previously Implemented De-labelling Program*

Over two distinct 2 week periods, the study member (physician) used a daily report generator on the *Epic* Electronic Medical Record system to identify all patients admitted to any medicine or surgery service at the hospital in the last 24 hours who were labeled with penicillin allergy. Patients were approached by the investigator after agreement by the patient’s most responsible physician (MRP). The investigator then used the Penicillin Allergy De-Labeling Algorithm reviewed by the Ottawa Hospital Antimicrobial Subcommittee to identify patients having a low-risk history of penicillin reaction (Version date 6 April 2021, supplemental figure 1). Inclusion criteria included all inpatients ≥ 18 years admitted for >24h to a medical or surgical service who had a reported penicillin allergy label listed in EPIC electronic medical record system were identified and screened by study personnel (physician). During participant screening, each patient’s record was reviewed to identify the presence of any exclusion criteria: a) pregnancy; b) respiratory or hemodynamic instability (SBP<100, HR>120, need for vasopressors, requiring > 4L/min oxygen); c) documented history of active suicidal ideation, dementia, current delirium or admission to psychiatry ward; d) active COVID-19 infection. The latter criterion was included for infection control reasons. Those with same-day surgical admission were excluded for the purposes of this study. Patients without exclusion criteria were approached by the assessor after agreement from the patient’s most responsible physician (MRP).

Those with any history suggestive of a severe, non Ig-E mediated reaction to penicillin (blistering, mucous membrane involvement, fever, joint pain/swelling) were told to continue to avoid penicillins. Patients with a high risk history of penicillin reaction (anaphylaxis, oropharyngeal angioedema, wheezing, hemodynamic alteration, any reaction within 1 year) were referred to Allergy and Immunology for further testing consideration. Similarly, patients with a moderate risk history of penicillin reaction (itching, hives, non-specific rash 1-5 years ago, time frame of reaction not clearly beyond this interval, any reported angioedema, recall of need for urgent medical attention) were referred to Allergy and Immunology.

Patients with a low risk history of penicillin reaction (cutaneous reaction >10 years ago, rash or doesn’t remember, no urgent medical attention needed, or patient does not know details of reaction occurring >5 years ago) underwent informed consent for oral challenge to a penicillin.

Patients with a family history of penicillin allergy but no personal history of a penicillin allergy or drug intolerance had their penicillin label directly removed from their chart. If patients could not be classified, using the above algorithm, they were reviewed at weekly meetings with investigator and board-certified Clinical Immunologist and Allergist to determine appropriate allergy testing group placement.

All patients electing to proceed with de-labelling provided verbal informed consent. The patients were then administered an oral challenge consisting of 250mg PO amoxicillin followed by 60 minutes of monitoring by direct supervision by the investigator on the hospital ward. Patients were monitored for adverse reactions including anaphylaxis. The occurrence of any of these symptoms or signs would have classified the patient as having had an adverse drug event. Patients without any of these findings were classified as ‘de-labelled patients’ and had their allergy status corrected on the electronic medical record system. In addition, a note was sent to their primary care provider and pharmacy regarding their corrected penicillin allergy status.
