# Supplementary material for: Introduction of a penicillin allergy de-labelling program with direct oral challenge and its effects on utilization of beta-lactam antimicrobials: a multicenter retrospective parallel cohort study
Source: Allergy Asthma Clin Immunol. 2024 Mar 5;20:20. doi: 10.1186/s13223-024-00877-9 (PMC10913637; doi:10.1186/s13223-024-00877-9)
Supplement: Supplementary file 2 — Additional file 2: Figure S1. Risk stratification algorithm. [file 13223_2024_877_MOESM2_ESM.docx]

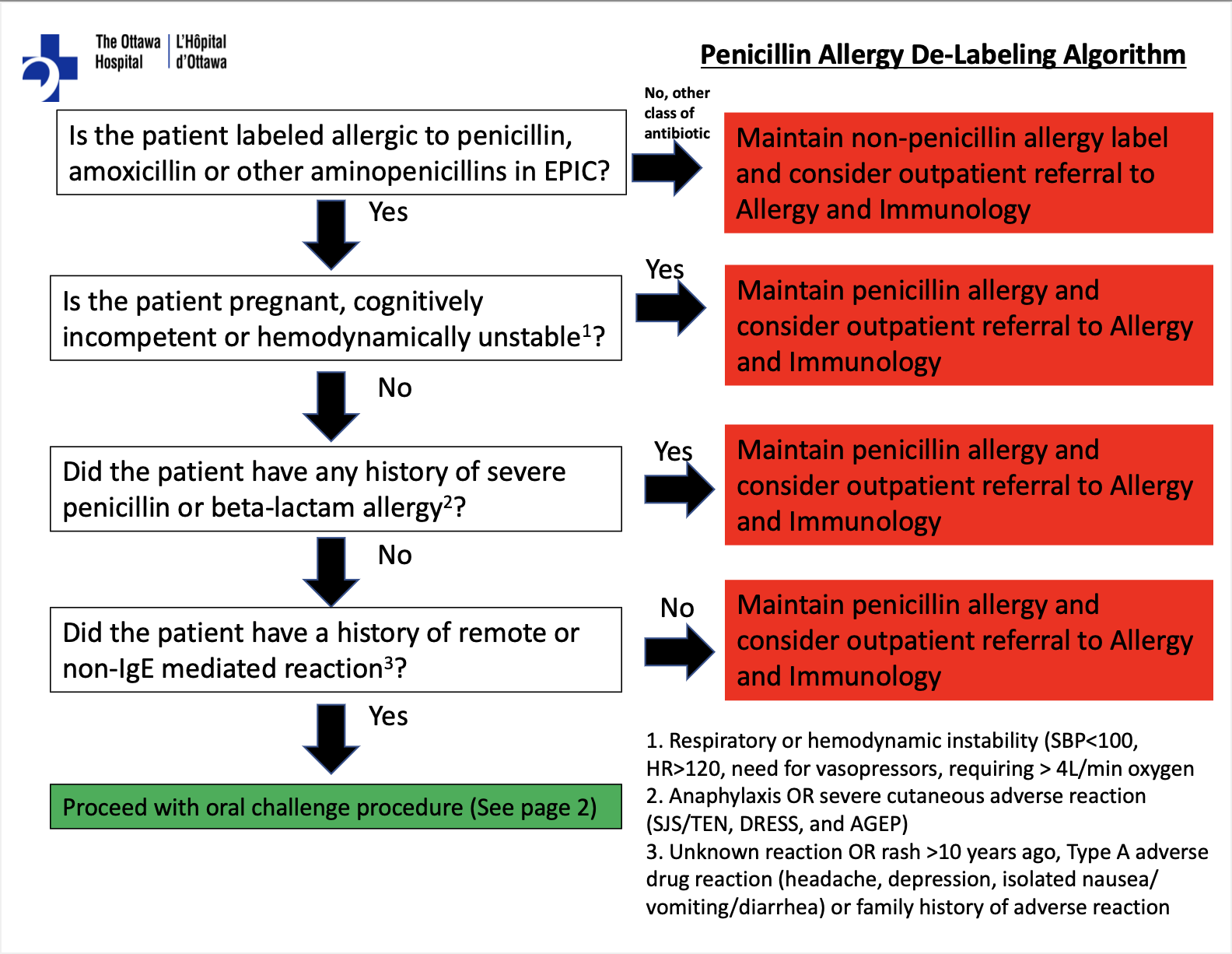


*Figure S1: Risk stratification algorithm*

*SJS: Steven-Johnson Syndrome, TEN: Toxic Epidermal Necrolysis, DRESS: Drug Rash with Eosinophillia and Systemic Symptoms, AGEP: Acute Generalized Exanthematous Pustulosis.*
